# The efficacy of dexmedetomidine for the prevention of catheter-related bladder discomfort: A systematic review and meta-analysis

**DOI:** 10.1097/MD.0000000000028217

**Published:** 2021-12-30

**Authors:** Jia Lu, Xiamin Yang, Jie Zhang, Yuelong Huang

**Affiliations:** a Department of Anesthesiology, Huashan Hospital North Affiliated to Fudan University, Shanghai, PR China; b Department of Anesthesiology, Huashan Hospital Affiliated to Fudan University, Shanghai, PR China; c Department of Spine, Zhuji affiliated hospital of Shaoxing University, Shaoxing, Zhejiang, PR China.

**Keywords:** catheter-related bladder discomfort, dexmedetomidine, meta-analysis, randomized clinical trials

## Abstract

**Background:**

The effective therapy to reduce postoperative catheter-related bladder discomfort (CRBD) remained unknown.

**Objective:**

We attempted to manage the systematic review and a meta-analysis to clarify the efficacy of dexmedetomidine (DEX) in potential prevention on CRBD.

**Methods:**

We performed the meta-analysis on randomized clinical trials (RCTs), and searched the databases from Web of Sciences, Embase and referred Cochrane Library published from October 2016 to September 2020. Data extraction was carefully conducted by 2 authors, respectively. Meta-analysis that was applied synthetically concerns the incidence and severity of CRBD and the treatment effect of DEX on CRBD.

**Results:**

We acquired 5 RCTs with interventions of DEX on CRBD. Meta-analysis showed DEX has significantly reduced the incidence and severity of CRBD compared with control at 0 hour (risk ratios [RR] = 0.40, 95% CI = 0.53–0.29, *P* < .01), 1 hour (RR = 0.44, 95% CI = 0.34–0.57, *P* < .01), and 2 hours (RR = 0.43, 95% CI = 0.32–0.58, *P* < .01) and 6 hours (RR = 0.43, 95% CI = 0.29–0.63, *P* < .01). DEX was also associated with lower incidence of moderate to severe CRBD at 0, 1, and 6 hours after surgery. There were no significant differences in adverse events other than bradycardia, hypotension, and hypertension.

**Conclusion:**

The 5 RCTs showed great effectiveness in reducing the incidence and severity of the early and later postoperative CRBD. Meta-analysis showed that DEX interventions were useful in preventing the early and later postoperative CRBD without significant side effects.

## 1 Introduction

Lots of patients suffer from catheter-related bladder discomfort (CRBD) who undergoing bladder catheterization during the operative period. Studies have shown that more than 80% of patients experience mild CRBD, and about 27% to 55% of patients go through moderate or severe CRBD.[^1^,^2^] The characteristics of CRBD including uncomfortable sensation in the suprapubic region or a strong intention to micturate and even can not help themselves to pull out of the catheter. The appearance of CRBD is extremely disturbing to patients and increasing the postoperative complication. The mechanism underlying the CRBD is not quite clear, which may are caused by involuntary contractions of the bladder mediated by muscarinic receptors (especially M3).[^3^,^4^] Therefore, CRBD is one major cause of postoperative agitation and the strategy is urgently needed for the improvement of clinical approaches on CRBD.^[5]^

Various drugs have been administrated to treat CRBD. Dexmedetomidine (DEX) application intraoperative has testified to reduce the incidence and severity of CRBD without significant side effects.^[5]^ DEX, a highly selective α2-adrenore agonist, with analgesic, sedative, anxiolysis, sympatholytic, and sedative properties, is a very useful associated agent for general anesthesia. Moreover, DEX has shown antimuscarinic activity (M3 receptor) and it reduces bladder contractility via inhibiting the M3 receptor to prevent CRBD.^[6]^ Hence, the potential of DEX to therapy in CRBD need to further explore.

In the present study, we conducted a review and meta-analysis to evaluate the efficacy of DEX in preventing or reducing the incidence and severity of CRBD in the early and late postoperative period.

## 2 Methods

### 2.1 Literature search strategy

This systematic review was performed according to the Preferred Reporting Items for Systematic Reviews and Meta-analyses (PRISMA) statement.[^7^,^8^] Neither a special ethics review nor an ethical approval was necessary in the present review. Two authors independently searched databases from Web of Sciences, Embase, and referred Cochrane Library published from October 2016 to September 2020 which were randomized clinical trials (RCTs). A retrieved citations was performed based on the following subject terms: CRBD and DEX. A total of 68 articles were returned in the search strategy.

### 2.2 Selection criteria

Previous studies were included in the systematic review if they met all the pre-established criteria:

1. RCTs with DEX to prevent CRBD;
2. adult patients more than 18 years of age;
3. patients undergoing the operation general anesthesia.

We eliminated studies if they satisfied the exclusion criteria:

1. patients with different urinary catheterization;
2. patients undergoing surgery without general anesthesia;
3. patients including nonsurgical treatments; and
4. patients with an allergic history to DEX.

### 2.3 Data extraction

All data were extracted from eligible studies according the flow diagram by 2 reviewers (A and B) independently. Any disagreements was resolved by consultation with a third reviewer (C). The following information was extracted from each study: first author's name, publication year, study design, participant characteristics (gender, age), type of surgery, dose, and route of dexmedetomidine and incidence of CRBD. Among the severity incidence, the incidences of mild to severe CRBD at 0, 1, 2, 4, and 6 hours after the surgery were extracted. If the severity incidence were not directly given, data in tables or figures were extracted according to the method described by Agarwal.^[5]^

### 2.4 Statistical analysis

The present study mainly focused on the incidence of CRBD and the relevant treatment strategy, which was performed for 5 articles using the same reagents. All the variables between 2 groups were presented as risk ratios (RRs) with 95% confidence intervals (CIs) without significant heterogeneity (*P* value of X^2^ test <0.10 and *I* ^2^ > 50%). The publication bias and studies quality were assessed in RevMan 5.3 software (RevMan, The Cochrane Collaboration, Oxford, UK). All *P* values were 2 sided, and a *P* value of less than .05 was considered statistically significant.

## 3 Results

### 3.1 Study characteristics

**Figure 1:**
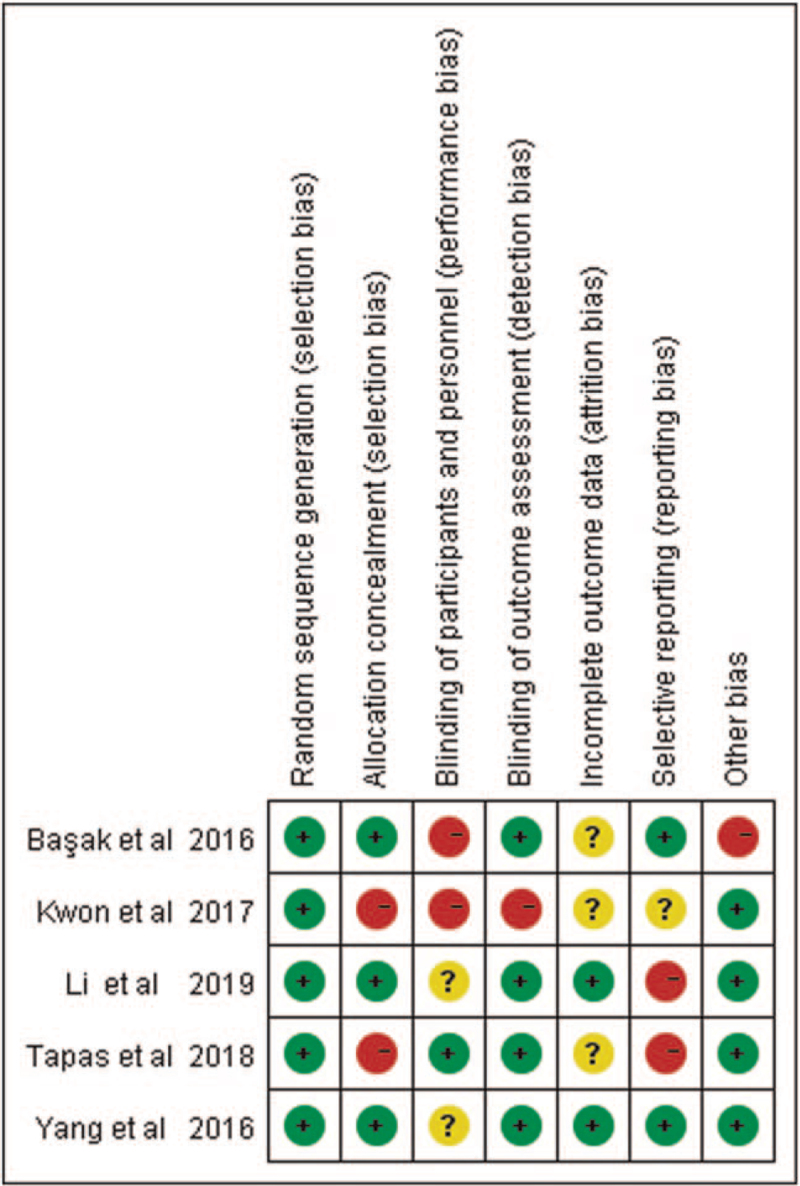
Risk of bias summary. Reviewing authors’ judgments about each risk of bias item for 5 eligible articles.

**Figure 2:**
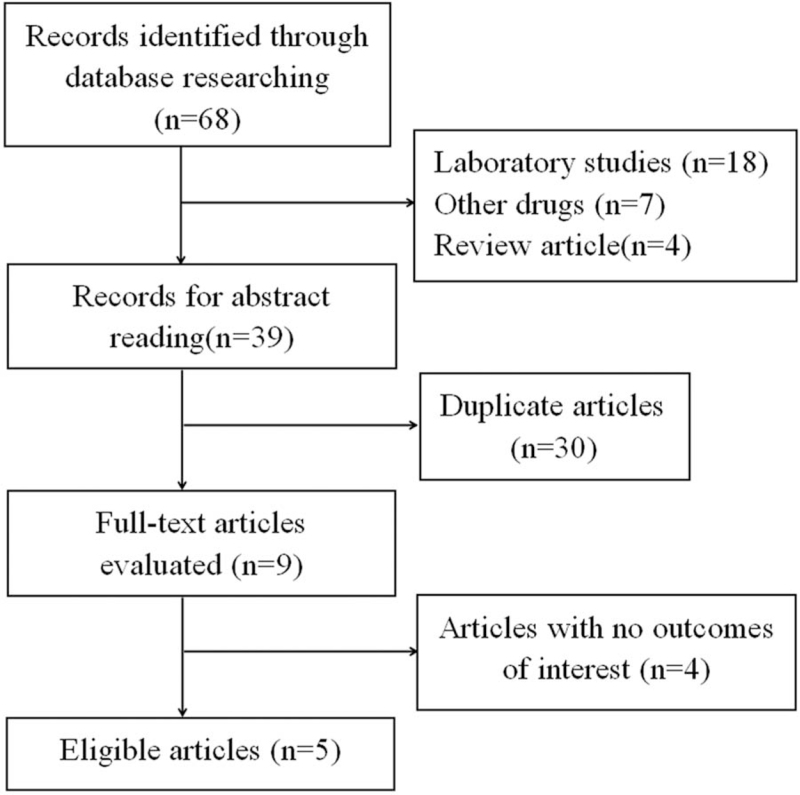
Flow diagram of the inclusion and exclusion processes.

Our study systematically search of PubMed, Web of Sciences, Embase and yielded 68 articles potentially met the inclusion criteria. After retrieval and review of the titles and abstracts by 2 authors independently, we removed 29 unqualified articles. Then, a total of 30 duplications were excluded. The remaining 9 articles were examined in detail and studies that had no outcomes of interest (n = 4) were excluded as well. Thus, 5 eligible articles fulfilled the criteria for meta-analysis. An overview of the risk of bias was shown in Figure 1. The all selection processes were shown in Figure 2 and the characteristics of 5 included articles were summarized in Table 1.

**Table 1:** The basic characteristics of included studies.

Study	Year	Total patients	Gender	Study design	Catheter size	Anesthesiology	Type of surgery	Dose	Duration operation(m)
Yang et al	2016	138	Either	RCT	16Fr	General	Abdominal surgery	0.5 μg/kg	164 ± 28
Tapaset al ^[19]^	2018	100	Either	RCT	16Fr	General	Kidney surgery	0.4 μg/kg	NG
Kwon et al	2017	70	Either	RCT	16Fr	General	Lumbar surgery	0.3-0.5 μg/kg	101 ± 20
Li et al^[20]^	2019	120	Either	RCT	16Fr	General	Hysterectomy	1.0 μg/kg	124 ± 31
Başak et al ^[21]^	2016	75	Either	RCT	NG	General	Cystoscopy	0.5 μg/kg	NG

A total of 5 articles with 503 patients were enrolled in the review and meta-analysis. The incidence of CRBD was significantly lower in group D (DEX group) than in group C (control group) at 1, 2, 4, and 6 hours, respectively. The severity of CRBD (mild, moderate, and severe) among groups were similar (*P* > .05) and were decreased in DEX group at all-time points (*P* < .05) evaluated in this study (Table 2).

**Table 2:** Incidence and severity of CRBD in different articles.

		T0			T1			T2			T6		
Study	Group	M	M1	S	M	M1	S	M	M1	S	M	M1	S
Yang et al	C	19	25	7	21	20	6	22	15	4	21	11	1
(n = 69)	D	10	6	0	11	3	0	11	3	0	13	2	0
Tapas et al	C	4	11	6	^∗^	^∗^	^∗^	5	14	2	^∗^	^∗^	^∗^
(n = 50)	D	3	5	1	^∗^	^∗^	^∗^	2	5	2	^∗^	^∗^	^∗^
Kwon et al	C	^∗^	^∗^	^∗^	5	13	4	^∗^	^∗^	^∗^	6	12	1
(n = 35)	D	^∗^	^∗^	^∗^	7	4	1	^∗^	^∗^	^∗^	5	1	0
Li etal	C	4	7	4	7	6	2	7	4	1	^∗^	^∗^	^∗^
(n = 25)	D	8	5	4	5	2	1	5	2	1	^∗^	^∗^	^∗^
Başak et al	C	9	13	4	9	17	2	13	10	0	9	0	0
(n = 39)	D	7	6	0	6	9	0	6	5	0	5	0	0

In this study, we systematically summarized the effect of DEX on the incidence of CRBD. We identified 5 articles with 436 patients concerning the CRBD to compare the DEX with the placebo. There was no significant differences in adverse events other than bradycardia and hypotension in group D (Table 3).

**Table 3:** Incidence of adverse effects.

	Group C	Group D	*P* value
Number	218	218	
Hypotension	5	19	< .05
Bradycardia	4	26	< .05
Hypertension	5	7	> .05
Nausea and vomiting	0	2	> .05
Others	0	0	> .05

**Figure 3:**
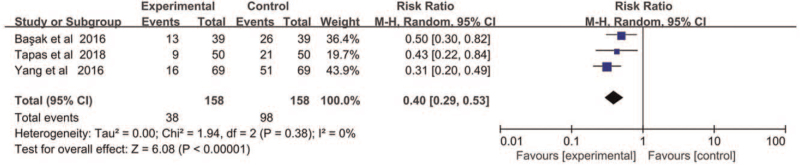
The incidence of CRBD at 0 hour after operation.

We carried out meta-analysis with 3 articles at 0 hour after operation. No heterogeneity between studies was observed, and therefore fixed-effect model (X^2^ = 1.94, *P* < .01, *I* ^2^ = 0%) was applied in the meta-analysis at 0 hour. The result demonstrated that DEX decreased the incidence of CRBD significantly (RR = 0.40, 95% CI = 0.53–0.29, *P* < .01) (Fig. 3).

**Figure 4:**
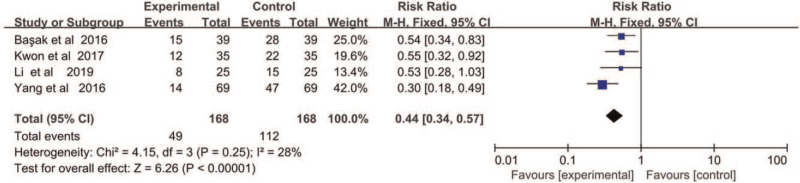
The incidence of CRBD at 1 hour after operation.

Four studies described the effect of DEX on CRBD at 1 hour after operation. There were no heterogeneity between studies. Furthermore, meta-analysis using fixed effect model (X^2^ = 4.15, *P* = .25, I^2^ = 28%) demonstrated that DEX reduced the incidence of CRBD significantly (RR = 0.44, 95% CI = 0.34 – 0.57, *P* < .01) at 1 hour after operation (Fig. 4).

**Figure 5:**
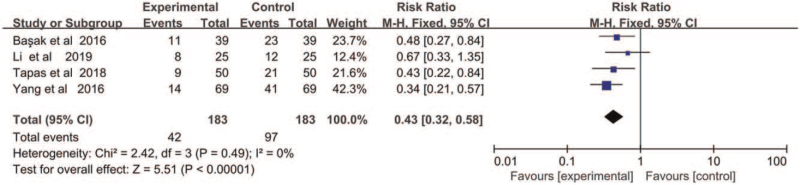
The incidence of CRBD at 2 hours after operation.

Three studies using DEX drugs concerning the effect on CRBD with 183 patients were included for meta-analysis. Meta-analysis using fixed effect model (X^2^ = 2.42, *P* = .49, *I* ^2^ = 0%) found that DEX significantly reduced the incidence of CRBD (RR = 0.44, 95% CI = 0.32–0.58, *P* < .01) comparing with control group at 2 hours after the postanesthesia care (Fig. 5).

**Figure 6:**
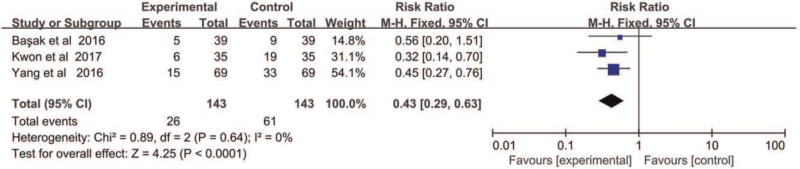
The incidence of CRBD at 6 hours after operation.

We performed meta-analysis on 3 articles choosing DEX as the treatment strategy. There were no heterogeneity in those studies. Hence, the fixed effect model (X^2^ = 0.89, *P* = .64, *I* ^2^ = 0%) was applied and the result demonstrated that 3 was significantly difference (RR = 0.43, 95% CI = 0.29–0.63, *P* < .01) between DEX group and placebo group at 6 hours (Fig. 6).

## 4 Discussion

CRBD, characterized by a discomfort feeling in the suprapubic region or an urge to micturate who have underwent urinary catheter during the operative period. The incidence and severity of CRBD after operation is ambiguous according to the studies.[^9^,^10^,^11^,^12^] According to the previous studies, we know that CRBD can lead to postoperative agitation, thus affecting the quality of resuscitation after general anesthesia and increasing the postoperative complications. Meanwhile, CRBD was treated as a recognized risk factor for postoperative emergence agitation.^[13]^ Despite various treatment options for CRBD, there are no specific clinical experiences and ideal agent to manage CRBD is absent to improve the satisfaction of patients. Therefore, the effective strategy of DEX treatment for prevention of CRBD should be figured out and demonstrated urgently.

We performed a systematic meta-analysis including 5 RCTs with interventions to evaluate the efficacy of DEX application for the prevention of CRBD. In our present systematic review, there were no differences between total patients, genders, the Foley catheter, and duration operation. Our study shows that DEX can decrease the incidence of CRBD significantly at 0, 1, 2, and 6 hours. Still, it can reduce the severity levels of CRBD at 0, 1, and 6 hours after surgery without causing severe drug-related adverse events as compare to the control group. Recent study has revealed that DEX was the relatively effective for reducing the overall appearance of CRBD.^[14]^ These results synthetically demonstrated that DEX could be a drug worthy of recommendation for the prevention of CRBD.

The main mechanisms of CRBD are probably due to involuntary bladder contraction mediated by muscarinic receptors(M-Rs) and bladder spasm by stimulation of the mucous membrane of urinary tract.^[15]^ Although CRBD was also found to be mediated by local inflammation with activation of the cyclooxygenase pathway and release of prostaglandin.^[21]^ The symptoms of CRBD are known to be related to types 2 and 3 (M2 and M3 receptors) among the 5 types of muscarinic receptors.^[16]^ An urinary catheter stimulates the afferent nerve of the bladder, and then releases the neurotransmitter acetylcholine. DEX is a highly selective alpher-2 adrenergic agonist, which have the function of analgesic, sympatholytic, and sedative. Thus dexmedetomidine can reduce bladder constraction via inhibiting M3 receptors and agonist alpha -2 receptors, which are widely distributed both the central and peripheral nervous systems.[^5^,^17^,^18^] Our study showed the similar results that DEX decrease the incidence and severity of CRBD, which proved that the drug acts by M3 receptors and agonist alpha-2 receptors.[^17^,^18^] Therefore, DEX might have contributed to decreasing the incidence and alleviating the severity of CRBD by the combination on the peripheral and central nervous system. However, hypotension and bradycardia are the most common adverse effects of DEX that should be concerned and that was showed in our meta-analysis.^[17]^

There also had some limitations in our systematic review and meta-analysis. First, the type of surgery in the included studies were various, which may influence the homogeneity of the study. Second, all included studies had not showed the all-time point that reduced the number of statistics and affected the validity of results.

## 5 Conclusion

The present systematic review and meta-analysis had shown that DEX have great effectiveness in reducing the incidence and severity of the early and later postoperative CRBD and were useful in preventing the early and later postoperative CRBD perioperatively.

## Author contributions

**Data curation:** Jia Lu, Yuelong Huang.

**Formal analysis:** Jia Lu, Xiamin Yang.

**Project administration:** Xiamin Yang, Jie Zhang.

**Supervision:** Jie Zhang, Yuelong Huang.

**Writing – original draft:** Yuelong Huang.

**Writing – review & editing:** Yuelong Huang.
